# Fingolimod Leads to Immediate Immunological Changes Within 6 h After First Administration

**DOI:** 10.3389/fneur.2020.00391

**Published:** 2020-05-12

**Authors:** Tony Sehr, Katja Akgün, Rocco Haase, Tjalf Ziemssen

**Affiliations:** Department of Neurology, Center of Clinical Neuroscience, University Hospital Dresden, Dresden, Germany

**Keywords:** multiple sclerosis, fingolimod (FTY720), autonomic, immunologic—flow cytometry, observational studies

## Abstract

**Objective:** Multiple effects of fingolimod have already been described. Here we investigated the acute effects on immune cell subsets and identified correlations with autonomic first dose phenomena and long-term immunological effects.

**Methods:** Blood samples of 20 MS patients were analyzed using FACS. Immune cell frequencies before and at defined prospective time points beginning 6 h after first fingolimod administration were evaluated in parallel to cardiovascular autonomic and clinical parameters.

**Results:** A significant decrease of absolute lymphocyte count (1.81GPt/l to 1.42GPt/l), CD3+ (1.34GPt/l to 1.06GPt/l), CD3+CD4+ (0.94GPt/l to 0.73GPt/l), and CD19+ (0.26GPt/l to 0.19GPt/l) cells could be already demonstrated within 6 hours after first dose which correspond to a relative reduction by 28, 23, 23% resp. 29% in relation to the longterm steady state cell frequency level. Short- and long-term effects were significantly correlated for lymphocytes, CD3+, CD3+CD4+, CD3+CD8+, CD19+, CD14+, and NK cells as well as for neutrophil granulocytes. In addition, correlations could be found between reduced heart rate (68.95–60.05 bpm) and the decrease in CD3+, CD3+CD4+, and CD19+ cells after 6 h.

**Conclusions:** Early immunological changes could already be detected 6 h after fingolimod first dose. Most of the acute changes correlate with long-term modulation. A link between the acute immunological and cardiological effects was found.

## Introduction

Fingolimod (FTY) has been approved for treatment of relapsing-remitting multiple sclerosis (RRMS) (1). As a structural analog to sphingosine-1-phosphate (S1P), it induces internalization of the S1P receptors (S1PR) on lymphocytes (2, 3). A pronounced lymphopenia in the peripheral blood develops (3, 4). A variety of studies investigated differential immunological changes due to FTY therapy (5, 6). Little information is available about acute effects within the first hours after first FTY intake. Here we evaluate immediate effects of FTY treatment on different peripheral immune cell subsets and set the results in context to the long-term changes.

In addition to peripheral lymphocyte subsets, S1PRs are expressed on different human tissues as on myocytes (1). That explains side effects including bradycardia. We performed extensive autonomic testing before and 6 h after start of FTY (7). The results were set in to investigate the link between cardiovascular-autonomic and immunological effects of FTY therapy. As well correlations to clinical parameters have been looked for.

## MethodS

### Patient Consent, Standard Protocol Approval and Registration

We analyzed EDTA blood and autonomic parameters of 20 patients diagnosed with RRMS according to the McDonald criteria 2010 (Table 1). All patients were treated following the actual German guideline recommendations. The study procedures were approved by the local ethical committee (EK 348092014). Informed written consent was obtained from all patients. As the immunological study reflects an observational study no registration as a public trail was applied. Autonomic parameters were obtained as part of the START study (NCT01585298).

**Table 1:** Patient characteristics.

**No**.	**Age range**	**BMI**	**Years MS**	**Previous treatment**	**ARR prev. year**	**Baseline EDSS**
1	30–34	20.2	12	Natalizumab	1	3.0
2	40–44	21.7	13	Glatirameracetat	3	3.0
3	45–49	20.0	1	β-1b-Interferon	1	2.0
4	45–49	26.9	6	β-1a-Interferon	1	2.0
5	40–44	30.5	10	Natalizumab	3	5.0
6	45–49	22.3	14	no	4	4.5
7	40–44	22.9	0	no	2	1.5
8	40–44	29.0	19	Natalizumab	3	2.5
9	25–29	20.3	10	β-1a-Interferon	0	2.5
10	25–29	18.4	10	β-1a-Interferon	1	6.0
11	25–29	20.5	3	β-1a-Interferon	2	3.5
12	40–44	32.3	3	Glatirameracetat	1	1.5
13	30–34	25.7	1	Glatirameracetat	0	3.0
14	40–44	27.2	9	β-1a-Interferon	1	2.5
15	40–44	22.8	2	no	2	4.0
16	25–29	13.2	3	β-1a-Interferon	3	2.0
17	50–54	21.0	5	β-1a-Interferon	3	4.5
18	40–44	26.5	1	β-1a-Interferon	0	2.0
19	40–44	29.4	9	β-1b-Interferon	1	2.0
20	40–44	29.7	2	no	1	2.0

*Characteristics of evaluated MS patients including age range (years), body mass index (BMI, kg/m^2^), years since MS diagnosis was met (years MS), previous treatment, annual relapse rate 12 months before baseline (ARR prev. year) and expanded disability status score (Baseline EDSS) at baseline (n = 20)*.

Two patients discontinued FTY therapy after month 4 resp. month 12 time point. One female MS patient (No. 9) got pregnant. One male MS patient (No. 11) developed secondary progressive disease course of MS.

### Immune Cell Phenotyping

The number of total leukocytes, lymphocytes, granulocytes and monocytes were measured on the Sysmex XN5000 automatic analyzer (Norderstedt, Germany).

Flow cytometry of immune cells from blood was performed as follows: 50 μl of total blood was subjected to erythrocyte lysis on a FACS Lyse Wash Assistant (Heidelberg, Germany). Upon washing the cells with a CellWASH buffer the remaining leukocytes were used for cell phenotype analysis. The analysis was performed on a FACS LSR II flow cytometer and visualized with the FACSDiva software. The following combination of antibodies (all from BD) was used: CD45-APC, CD3-APC-Cy7, CD19-PE-Cy7, CD4-PE-Cy7, CD16-FITC, CD56-FITC, CD8-PerCP.

### Autonomic Cardiovascular Monitoring

Different autonomic cardiovascular parameters such as heart rate, blood pressure, baroreflex sensitivity, total peripheral resistance, cardiac output and heart rate variability were evaluated up to 6 h after drug intake. Data have been collected as published elsewhere (8).

### Statistical Analysis

Data are expressed as mean and standard derivation. Wilcoxon matched pairs test was used to investigate differences between baseline and 6 h data. Friedman test was used for repeated measure testing with Dunn's post-test for pairwise comparisons. Correlations were analyzed using Spearman rank correlation. For correlation analysis between immunological short- and long-term effects, the ratio (6 h-BL)/BL and (Month12-BL)/BL was built.

## Results

### Immediate Immunological Effects After First Dose of FTY and Correlation Analysis

Six hours after first drug intake, we could demonstrate a significant decrease of absolute lymphocyte count (1.81–1.42 GPt/l), CD3+ (1.34–1.06 GPt/l), CD3+CD4+ (0.94–0.74 GPt/l), and CD19+ cells (0.26–0.19 GPt/l) (see Table 2A). These decreases correspond to a relative reduction of 28, 23, 23, and 29% in relation to long-term immunological study state after 12 months. CD14+ cells showed an absolute increase (0.48–0.64 GPt/l), though leukocytes (6.63–6.45 GPt/l), CD3+CD8+ (0.37–0.31 GPt/l), NK cells (0.16–0.18 GPt/l), and neutrophils (4.15–4.12 GPt/l) kept stable (see Table 2A).

**Table 2:** Short-term effects and correlation with long-term effects.

	**A**			**B**	
	**Cell count [GPt/l]**, ***n*** **=** **20**			**Correlation short-/long-term**, ***n*** **=** **19**	
	**BL**	**6 h**	***P*-value**	**Spearman r**	***P*-value**
Leukocyte	6.63	6.45	0.305	−0.140	0.568
Lymphocyte	1.81	1.42	0.007	0.423	0.007
CD3+	1.34	1.06	<0.001	0.498	0.030
CD3+CD4+	0.94	0.74	<0.001	0.553	0.014
CD3+CD8+	0.37	0.31	0.094	0.628	0.004
CD19+	0.26	0.19	<0.001	0.501	0.029
CD14+	0.48	0.64	<0.001	0.530	0.012
NK cell	0.16	0.18	0.658	0.613	0.005
Neutr. Gran.	4.15	4.12	0.920	0.535	0.018
	**Heart rate [bpm]**, ***n*** **=** **20**				
	**BL**	**6 h**	***P*****-value**		
	68.95	60.05	0.001		

*(A) Presented are cell counts of different immune cell populations and the heart rate at baseline (BL) and 6 h after start of FTY treatment. Wilcoxon matched pairs test was used to calculate the significance level of the differences (ns, not significant). (B) Presented are spearman r values and p-values as the results of the correlation analysis of the ratio of immunological short- (6 h to baseline) to long-term (month 12 to baseline) FTY treatment effects*.

We could demonstrate a significant link between the amount of the described immediate and long-term immunological changes (after 12 months) for lymphocytes (*r* = 0.423), CD3+ (*r* = 0.498), CD3+CD4+ (*r* = 0.014), CD3+CD8+ (*r* = 0.014), CD19+ (*r* = 0.029), CD14+ (*r* = 0.012), and NK cells (*r* = 0.005) as well as neutrophil granulocytes (*r* = 0.018) (see Table 2B) by correlation analysis.

### Autonomic Cardiovascular Effects After FTY Administration and Correlation With Immunological Effects

Within 6 h after FTY administration, the heart rate was significantly reduced (68.95–60.05 bpm) (see Table 2A). Other investigated autonomic parameters such as baroreflex sensitivity (8.11–8.32 ms/mmHg, *p* = 0.601), total peripheral resistance (1.08–1.00 MU, *p* = 0.383), cardiac output (89.91–87.63 ml, *p* = 0.965) and heart rate variability (relative low frequency power of HRV 56.72–49.86%, *p* = 0.127; relative high frequency power of HRV 25.13–33.77%) did not show a significant change. We did not observe any cardiovascular abnormality including blood pressure dysregulation or syncope. A 12 lead ECG demonstrated no relevant pathological abnormalities at 0 and 6 h.

Of all investigated parameters, only the correlation analysis between the decrease of the heart rate and the immediate decrease of CD3+ (*r* = 0.504, *p* = 0.024), CD3+CD4+ (*r* = 0.612, *p* = 0.004), and CD19+ cells (*r* = 0.638, *p* = 0.003) after 6 h was significant.

### Immunological Changes Within 24 Months FTY Treatment Period

**Figure 1:**
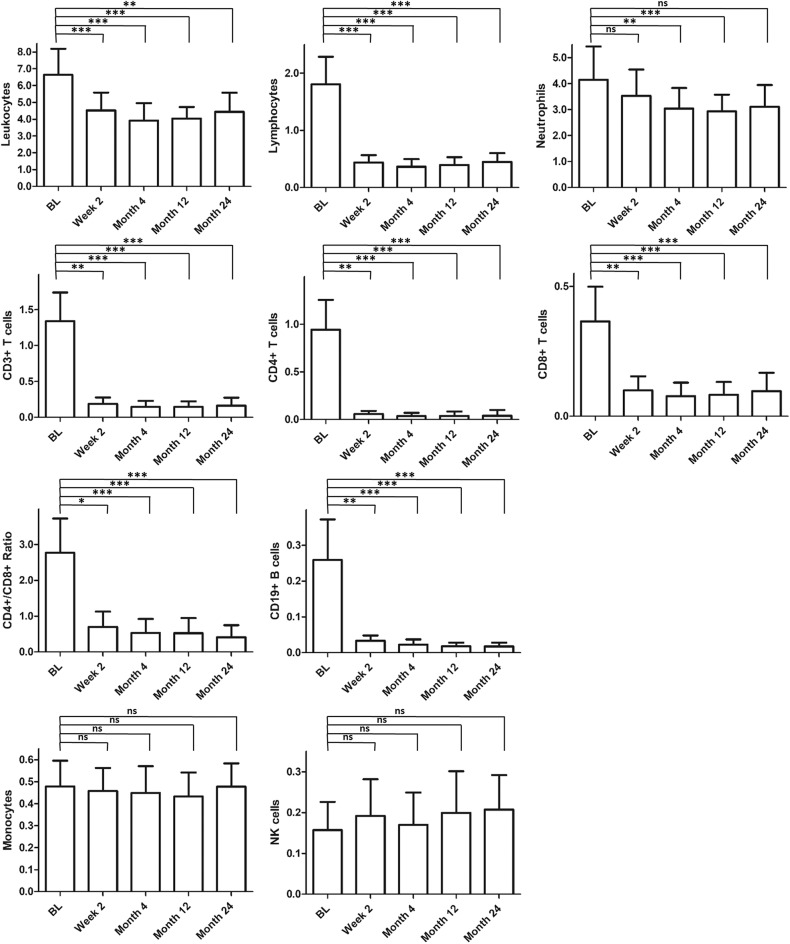
Long-term effect on immunological parameters. Figured are absolute cell counts [GPt/l] of different immune cell populations within observation period. Mean values and standard derivation at baseline (BL, *n* = 20) and different time points [2 weeks (*n* = 20), 4 months (*n* = 20), 12 months (*n* = 19), and 24 months (*n* = 18)] are depicted. For statistical analysis, Friedman test with Dunn's post-test was used. Asterisks indicate level of significance for pairwise comparison (ns, not significant, *p < 0.05, **p < 0.01, ***p < 0.001).

Over 24 months after treatment start, we could detect a significant decrease in total leukocytes and other immune cell populations (Figure 1). Because the CD3+CD8+ cell frequency was less affected, the CD4+/CD8+ ratio decreased. In addition, we found a significant decrease in neutrophil granulocytes. NK+ and CD14+ cells kept stable over therapy. The main changes of immune cell composition during FTY treatment reached a steady state 2 weeks after start of FTY treatment.

### Clinical Data and Correlation With Immunological and Cardiovascular Effects

All patients showed stable disease course without clinical relapses and MRI activity (no enhancing or new lesion) over the whole treatment period. No patient developed a severe lymphopenia below 0.2 GPt/l. No correlations could be detected between baseline clinical characteristics and immunological or cardiovascular changes.

## Discussion

The reduction of disease activity in MS by FTY is mainly driven by the reduction of auto-aggressive immune cells by inhibiting the recirculation out of lymph nodes. In this study, we evaluated the kinetics of immunological changes with special focus on immediate changes.

Via a distinct analysis of immune cells already 6 h after first drug intake, we could show that FTY-mediated functional antagonism of S1P-receptors with inhibition of cell egress from lymph nodes into blood is detectable within a few hours after first drug intake. Immune subsets were differentially affected as we have already reported (9). Lymphocytes, CD3+, CD3+CD4+, and CD19+ cells were significantly decreased, while leukocytes kept stable. As parts of the innate immune system, NK cells and neutrophil granulocytes kept stable. CD14+ cells were initially significantly increased and restored over the further treatment course. During ongoing FTY treatment, we found that 2 weeks after first FTY dose immune cell counts reach a steady state. The analysis of the immune cell composition after 2 weeks of FTY treatment could be suitable to get an impression of the total drug-mediated effects on lymphocyte distribution. Only neutrophil granulocytes showed a later decrease.

We could demonstrate a link between short- and long-term immunological treatment effects for most of the immune cell subsets. Interestingly this link could be demonstrated even for immune cell subsets which did not demonstrate significant changes in the first 6 h. Individual differences in CCR7 or S1PR expression patterns at baseline may explain these positive correlations. Our results emphasize the relevance of early changes to long-term immunological outcomes of FTY therapy. Immune cell counts 6 h after first FTY intake could be a prognostic marker for the development of treatment relevant lymphopenia <0.2GPt/l. Recently, Ohtani et al. found that the absolute lymphocyte count one day after FTY start predicted such lymphopenia with a sensitivity of 92% and a specificity of 76% (10).

In our study, we could replicate the well-known significant S1P-related adverse effect of heart rate decrease after initiation of FTY. Correlation analysis revealed an association between the degree of induced cardiological and immunological changes for several immune cell subsets. Ikeda et al. (11) have demonstrated a link between heart rate change and immunological effects as well. The explanation for this correlation could be related to an individual, but intraindividual comparable expression and composition of S1P-receptors between heart and immune cells. That would lead to an individually determined sensitivity to drug effects in different organ systems.

During the study period all examined patients presented a stable disease course. Additionally, cerebral imaging by MRI did not show relevant changes. Therefore, no correlations between different clinical response profiles and the investigated immunological or cardiovascular parameters could be detected.

The major limitation of our study is the small cohort size. Statistical analyses and conclusions would improve increasing sample size. As another limitation, we performed only a limited set of immunological analysis after FTY first dose. In this study we focused on the most relevant immune cell subsets including innate immune cells. Especially the detailed pharmacodynamic evaluation of the immunological changes within the first two weeks would be important.

Summarizing the results of our pilot study, we present significant immunological and cardiological changes in the first 6 h after FTY start, a strong correlation between short- and long-term immunological effects and an association between cardiological and immunological changes.

## Data Availability Statement

The authors take full responsibility for the data, the analyses and interpretation, and the conduct of the research. The datasets generated during and/or analyzed during the current study are available from the corresponding author on reasonable request.

## Ethics Statement

The studies involving human participants were reviewed and approved by Ethical committee of the Technical University of Dresden (EK 348092014). The patients/participants provided their written informed consent to participate in this study.

## Author Contributions

TS contributed to data curation, formal analysis, investigation, methodology, validation, visualization, and writing the original draft. RH contributed to formal analysis, methodology, and reviewing and editing the manuscript. KA contributed to investigation, methodology and reviewing and editing the manuscript. TZ contributed to conceptualization, investigation, project administration, supervision, validation, and reviewing and editing the manuscript.

## Conflict of Interest

The authors declare that the research was conducted in the absence of any commercial or financial relationships that could be construed as a potential conflict of interest.
